# Modeling Colorectal Cancer Progression Reveals Niche-Dependent Clonal Selection

**DOI:** 10.3390/cancers14174260

**Published:** 2022-08-31

**Authors:** Nuria Vaquero-Siguero, Nikolai Schleussner, Julia Volk, Manuel Mastel, Jasmin Meier, Rene Jackstadt

**Affiliations:** 1 Heidelberg Institute for Stem Cell Technology and Experimental Medicine (HI-STEM gGmbH), 69120 Heidelberg, Germany; 2 Cancer Progression and Metastasis Group, German Cancer Research Center (DKFZ) and DKFZ-ZMBH Alliance, 69120 Heidelberg, Germany; 3 Faculty of Biosciences, Heidelberg University, 69120 Heidelberg, Germany; 4 Department of General, Visceral and Transplantation Surgery, University Hospital Heidelberg, Heidelberg University, 69120 Heidelberg, Germany; 5 Faculty of Medicine, Heidelberg University, 69120 Heidelberg, Germany

**Keywords:** colorectal cancer, metastasis, mouse models, mouse-derived organoids, orthotopic transplantation, tumor heterogeneity, clonal selection, niche

## Abstract

**Simple Summary:**

Colorectal cancer (CRC) starts as a localized tumor and becomes a systemic disease with fatal consequences. However, clonal dynamics during progression are not well understood. Here, we present various techniques to model the different stages of CRC progression. Using genetically engineered mouse models (GEMMs) or organoid transplantation, localized tumors as well as liver metastases with characteristic intra-tumor heterogeneity were generated. The optical barcoding of transplanted organoids revealed niche-dependent clonal selection, implying that distinct niche factors control clonal outgrowth.

**Abstract:**

Colorectal cancer (CRC) is among the deadliest cancers worldwide, with metastasis being the main cause of patient mortality. During CRC progression the complex tumor ecosystem changes in its composition at virtually every stage. However, clonal dynamics and associated niche-dependencies at these stages are unknown. Hence, it is of importance to utilize models that faithfully recapitulate human CRC to define its clonal dynamics. We used an optical barcoding approach in mouse-derived organoids (MDOs) that revealed niche-dependent clonal selection. Our findings highlight that clonal selection is controlled by a site-specific niche, which critically contributes to cancer heterogeneity and has implications for therapeutic intervention.

## 1. Introduction

With a continuously rising incidence and more than 900,000 associated deaths worldwide per year, CRC is one of the main contributors to cancer related fatality [1]. In women and men, it is the second and third most common cancer type, respectively [1]. Furthermore, the incidence is predicted to rise beyond 2.5 million cases per year by 2035 [2]. While lifestyle changes can significantly contribute to reduce CRC onset, complex screening programs are currently the best options for decreasing CRC incidence [3]. Patient survival is dependent on the stage at diagnosis, as localized tumors (stage I–II) have a better prognosis than metastatic CRC (stage IV) [4]. Indeed, the 5-year overall survival rate drops drastically from ~64% for patients diagnosed in stage I–II to 12% for patients diagnosed in stage IV [4]. The standard treatment for CRC in stage I–III is the surgical removal of the primary tumor, after which up to 65% of the patients are cured [5]. However, systemic disease remains the leading problem, as the clinical management of advanced CRC is hampered by treatment resistance or recurrence of initially responsive tumors. As a consequence, ~90% of CRC related deaths are associated with metastatic disease that does not response to currently available systemic treatments [1]. The clonal evolution of cancer cells drives reduced therapeutic efficacy at later stages when the disease has progressed [6]. Importantly, this constant tumor adaptation to changes in the environment at the primary or secondary site is a major challenge when treating CRC [7]. Therefore, it is crucial to recapitulate the multifactorial process during the metastatic cascade in faithful models to consider for example niche-dependent regulatory mechanisms. This includes all steps of the invasion–metastasis cascade, from invasion to intravasation, overcoming stresses in the bloodstream and extravasation at secondary organs. Hence, models that resemble the anatomical, molecular, and histopathological characteristics of human CRC are crucial to better understand the mechanism of metastasis.

A wide range of GEMMs and patient-derived xenografts (PDXs) are the gold standard to model CRC and have been used for decades to analyze human CRC [8,9]. However, these models still bear a number of limitations. While tumor initiation is driven by genes that are frequently altered in human CRC, they often generate anatomically imprecise tumors with the highest burden in the small intestine instead of the large intestine [8]. Furthermore, models that robustly simulate the entire invasion–metastasis cascade with high metastatic propensity are lacking. We recently developed models that meet these criteria and improve the CRC model landscape. For instance, to precisely generate right-sided tumors that are associated with poor survival [10], we generated a mouse model with alterations in *Braf* and *Alk5* (encoding for TgfbrI). Resulting tumors were mainly located to the right-sided colon and showed similar to patients’ mucinous histopathology [11]. Further, the association of *APC* mutations to left-sided tumors rationalized the deletion of *Apc* via the intra-colonic injection of 4-hydroxytamoxifen (4-OHT), which led to efficient tumor initiation [12]. Hence, with local application of 4-OHT, the number of tumors can be controlled and tumor formation is achieved exclusively at the site of injection [12,13,14]. The preclinical use of this model was utilized to validate novel therapeutic strategies and demonstrated that BCL-XL inhibition causes impaired adenoma outgrowth [15].

Moreover, we generated a highly metastatic GEMM that is driven by alterations in the oncogene *Kras* (*Kras*^LSL-G12D/+^), the tumor suppressor *Trp53* (*p53*^fl/fl^), and activation of the Notch pathway (*Rosa26*^LSL-N1ICD/+^) (KPN) [16]. KPN mice generate spontaneous metastatic tumors with abundant stromal infiltration and transcriptional profiles reminiscent of the poor-prognosis CRC consensus molecular subtype (CMS) 4 [17] and the CRC cell intrinsic subtype (CRIS) B [18,19]. In this model of serrated CRC, we found that the expression of epithelial Notch1 rewires the tumor microenvironment (TME) to become immunosuppressive and metastasis-promoting [16].

In recent years, various advances have built on the advantages of GEMMs to model CRC [20]. The generation of normal intestinal organoids and tumor-derived organoids paved the way for a significantly increased relevance of in vitro systems [21]. When extracted from patient tumors or GEMM tumors, organoids as well as in vitro genetically engineered intestinal cells faithfully replicate the heterogeneity and molecular features of original tumors [16,22]. Importantly, transplanted organoids also establish the stem cell differentiation hierarchy, which is crucial to understanding the functional role of processes that contribute to intra-tumor heterogeneity, such as cancer stem cells and tumor cell plasticity during metastasis, therapy resistance or relapse [23,24,25].

Here, we used MDOs in versatile preclinical models to investigate clonal dynamics during CRC progression. We combined those immune-proficient models with an optical barcoding approach to demonstrate site-dependent clonal selection that highlights the enhanced relevance of syngeneic models to investigate metastasis and clonal dynamics of CRC.

## 2. Materials and Methods

### 2.1. Animal Models

The transgenic mouse lines used in the study were previously published: *villin*Cre^ERT2^ [26], *Apc* floxed [27], and *Trp53* floxed [28]. All animal experiments were performed according to the local ethics and animal experiment requirements. Mice were kept according to the local and latest standards of animal laboratory care. The tumor number was counted, the tumor size was measured, and the tumor volume was estimated using the following formula (length × width^2^)/2.

### 2.2. Production of Lentivirus

High-titer lentivirus production and purification were performed as follows. HEK293T cells were cultured in DMEM, 10% FBS in a humidified cell culture incubator with 5% CO_2_ at 37 °C. At sub-confluency, cells were transfected with a transfer plasmid (pCDH-EF1-Luc2-P2A-tdTomato, Addgene #72486; LeGO-GFP, Addgene #25917; LeGO-V2, Addgene #2734; LeGO-S2, Addgene #85211; LeGO-mOrange2, Addgene #85212; or LeGO-dKatushka2, Addgene #85214), as well as with the packaging plasmids (psPAX; Addgene #12260 and pMD2.D Addgene #12259) using transfection-grade linear polyethylenimine with a molecular weight of 25,000 Da (Polysciences #23966-1). The media were changed 12–24 h after transfection. 50 to 70 h after transfection, the supernatant containing the lentiviral particles was harvested and filtered. The lentivirus was concentrated by ultracentrifugation at 25,000 r.p.m. for 2 h at 4 °C (Optima L-90K Ultracentrifuge; Beckman Coulter, Krefeld, Germany) with a SW 32 Ti rotor (Beckman Coulter). Concentrated virus was re-suspended in Dulbecco’s modified eagle medium (DMEM) or phosphate buffered saline (PBS).

### 2.3. Culturing Organoids

Murine organoids were routinely cultured and passaged as previously described [16]. In brief, Cultrex reduced growth factor basement membrane matrix (BME; R&D Systems, 3433-005-01) domes containing organoids were rinsed with 4 °C cold PBS to detach the BME. After washing with 5 mL 4 °C cold PBS, samples were centrifuged at 450× *g* for 5 min. The supernatant was aspirated leaving a BME pellet and organoids. To break up the organoids, 150 µL of 4 °C cold PBS was added and pipetted vigorously and repeatedly. Subsequently, 5 mL of PBS was added and centrifuged at 300× *g* for 3 min. The supernatant was aspirated and organoids were suspended in BME (20 µL per dome) and plated on pre-warmed plates. Organoids were split at a ratio of 1:2 or 1:3.

The organoids were maintained in advanced DMEM/F12 (Gibco, 12634028) with 1% penicillin–streptomycin (Sigma, P4458-100ML), 1% D-Glutamine (Gibco, 25030024), 1% HEPES (Sigma, h0887-100), 1× B27 (Gibco, 12587010), and 1× N2 (Gibco, 17502048). KPN organoids were additionally supplemented with 100 ng/mL of Noggin (Peprotech, 250-38-100).

### 2.4. Organoid Transduction and Selection

Organoids embedded in BME were transferred into a centrifugation tube and dissociated mechanically by pipetting or by enzymatical digestion with 1× TrypLE Express (Invitrogen, Waltham, MA, USA, 12605010). Dissociated organoids were re-suspended in culture media supplemented with 8 µg/mL of polybrene (Sigma-Aldrich, St. Louis, MO, USA, TR-1003) and transferred to an ultra-low attachment 96-well plate. The desired volume of lentivirus solution was added to the well containing organoids and the plate was centrifuged at 1000 r.p.m. for 1 h at 30 °C (spinculation) using a Beckman Coulter table top centrifuge. For the transduction with the LeGO virus, organoids were separately infected with LeGO viruses. After centrifugation, the plate was incubated for 4–6 h at 37 °C. The virus–cell mix was washed with PBS, embedded in BME, and incubated at 37 °C until the BME was solidified and culture medium was added.

To select labelled organoids expressing the fluorescent proteins, organoids embedded in BME were dissociated into single cells with 1× TrypLE Express for 5 min at room temperature. Single cells were re-suspended in PBS with 1% FCS and passed through a 40 µm strainer. Color-positive cells were enriched using a BD FACSAria cell sorting system (Table 1). After centrifugation (300× *g*, 5 min at room temperature), cells were re-suspended in BME and plated.

Bright field and fluorescent images of the organoids were taken with a Nikon Eclipse Ti and a Zeiss LSM 710 and were processed with FIJI (ImageJ) software.

### 2.5. Flow Cytometry Analysis

Murine organoids from the in vitro competition assay were dissociated into single cells, as described above. Tumors were enzymatically dissociated (Tumor Dissociation Kit, Miltenyi 130-095-929) and passed through a 100 µm strainer. Single cells were incubated with ZombieNIR for 10 min at room temperature. The optical barcoded organoids and tumors were analyzed on a BD LSR Fortessa with a filter configuration listed in Table 2. The frequency of each clone was defined from the viable cell population.

### 2.6. Murine Colonoscopy-Guided Mucosal Injection

Murine colonoscopy was conducted using a Karl Storz TELE PACK VET X LED endoscope video unit or a Karl Storz endoscope system consisting of a suction and irrigation device (Karl Storz, vetpump2), documentation system (Karl Storz, AIDA), cold light source (Karl Storz, xenon 175), imaging system (image1 S, Karl Storz, Tuttlingen, Germany), and Hopkins Telescope (part 67030 BA). Mice were anaesthetized with isoflurane and 4-hydroxytamoxifen (4-OHT) or organoids were injected using a syringe (Hamilton Inc., Reno, NV, USA, 7656-01), a transfer needle to take up substances (Hamilton Inc., 7770-02), and an injection needle (Hamilton Inc., 7803-05). The injection needle was placed on the colon mucosa with the bevel facing the mucosa. Afterwards, 70 µL of 4-OHT or organoids was injected into the mucosa to form a bubble. Mice were sacrificed at week 6 or when endpoint was reached.

### 2.7. Intrasplenic Injection

To prepare single-cell suspensions, organoids were harvested, washed with PBS, and dissociated into single cells with 1× TrypLE Express (Gibco, 12605010) for 5 min at room temperature. TrypLE Express activity was quenched with PBS and cells were passed through a 40 µm strainer to separate single cells from fragments. 5 × 10^5^ single cells were injected per animal in 50 µL of PBS. For the transplantation of the optical barcoded KPN clones, an equal mix of the 21 color-barcoded clones was prepared.

C57BL/6 mice (6–12 weeks old males; Janvier Labs, Saint-Berthevin, France) were anesthetized with ketamine, xylazin, lidocaine, bupivacaine, and carprofen. The left side of the abdomen was shaved and a small incision on the flank of the mouse was carried out to expose the pancreas and spleen. The pancreas was gently pulled out of the abdominal cavity to access the spleen. An equal mix of the 21 optical barcoded KPN clones or parental KPN MDOs were injected into the spleen with a 25 G needle. After the injection, the pancreas and the spleen were placed back into the abdominal cavity and the incision was sutured. Mice were sacrificed after 4 weeks and liver metastases were dissociated as described before.

### 2.8. Sub-Cutaneous Injection and Tumor Monitoring

An equal mix of the 21 optical barcoded KPN clones was prepared and 5 × 10^5^ single cells in a final volume of 50 µL were injected in the left flank of C57BL/6 mice (6–12 weeks old males; Janvier Labs, Saint-Berthevin, France). The tumor growth was monitored 3 times per week and the animals were sacrificed when the tumor reached 1.5 cm or when an ulcer was observed. Tumors were dissociated as described above.

### 2.9. Histology

Organoids were prepared for histology by scraping the BME domes of at least two wells of a 6-well plate with a spatula and placed on Tissue-Tek filter paper (Vogel, SA-4699) and sequentially into a histology cassette. Both tissue and organoids were fixed using neutral buffered 10% formalin solution (Sigma, ht501128-4l) and processed according to standard histology processing techniques to generate paraffin-embedded tissue. Images were acquired using a 10× or 20× magnification on a Zeiss Axioscan.Z1 slide scanner or Zeiss Cell Observer and processed with FIJI (ImageJ) or ZEN software.

### 2.10. Immunofluorescence

Immunofluorescence was performed on 5 µm sections. Antigen retrieval was carried out with boiling target retrieval solution (Dako, S169984) for 30 min, and samples were blocked in TNB buffer (0.1 M Tris-HCL, pH 7.5, and 0.15 M NaCl with 0.5% *w*/*v* blocking reagent (Perkin Elmer, Boston, MA, USA, FP1020)) for 1 h at room temperature. Samples were incubated with primary antibodies against cytokeratin 20 (1:100; Abcam; AB52460) for 1 h at room temperature or mouse anti-β-catenin (1:100; BD Transduction Laboratories™, 610154) overnight at 4 °C. Alexa Fluor 488-conjugated (Life Technologies, goat anti-rabbit antibody, A11034) or Alexa Fluor 568 (A11034; Goat anti-Mouse IgG (H + L), A-11031) secondary antibody and DAPI were added for 30 min at room temperature, and slides were mounted with ProLong Gold Antifade Mountant (ThermoFischer Scientific, P10144). All images were taken with a Zeiss LSM 710 microscope and processed with FIJI (ImageJ) or ZEN software.

### 2.11. Immunohistochemistry

Immunohistochemistry (IHC) was performed on 5 µm sections. Antigen retrieval was carried out with boiling target retrieval solution (Dako, S169984) for 30 min, and samples were blocked in TNB buffer (0.1 M Tris-HCL, pH 7.5, and 0.15 M NaCl with 0.5% *w*/*v* blocking reagent (Perkin Elmer, FP1020)) for 1 h at room temperature. Samples were incubated with primary antibodies against Ki67 (Sigma, 275R-18) for 1 h at room temperature. Corresponding anti-rabbit biotinylated secondary antibodies and the ABC avidin–biotin–DAB detection kit (Vector laboratories, PK-6100) were used according to manufacturer’s instructions. Sections were counterstained with Mayer’s hematoxylin solution (Sigma-Aldrich, 51725) for 1 min, dehydrated using increasing concentrations of ethanol, and mounted with Cytoseal XYL (ThermoFisher Scientific, 12502736). Images were acquired using a 20× magnification on TissueFAX system (Tissuegnostic) and processed with FIJI (ImageJ) or ZEN software.

## 3. Results

### 3.1. Versatile Application of Orthotopic Colonoscopy-Guided Injections

**Figure 1:**
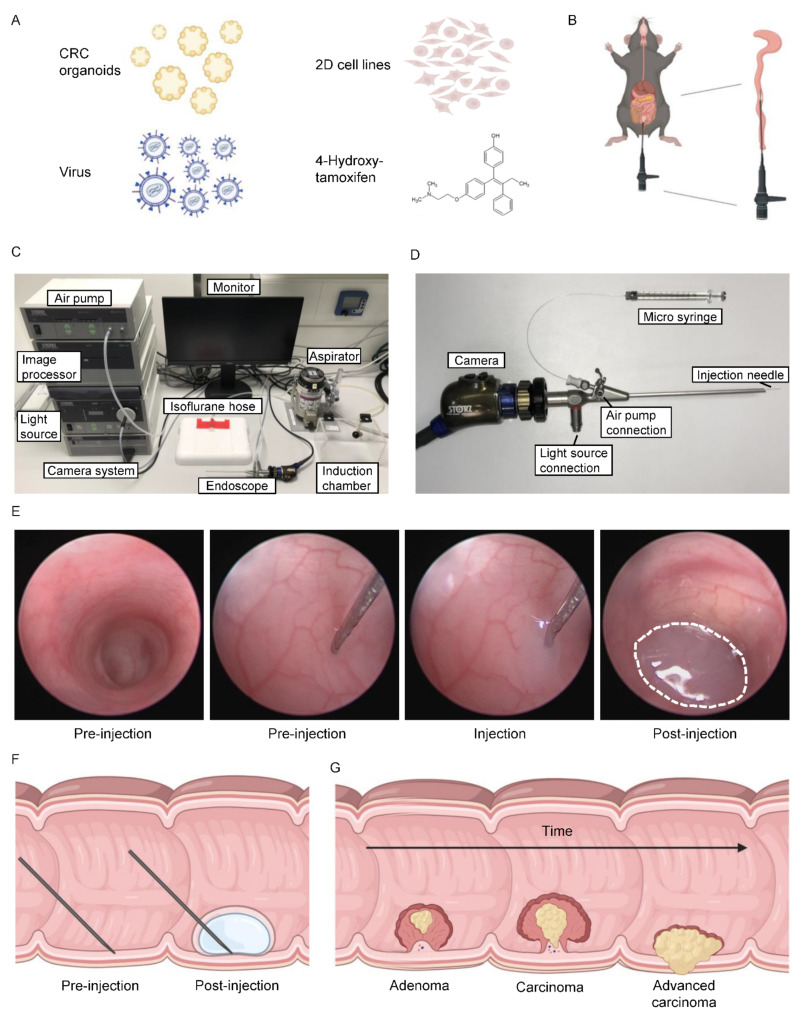
Colonoscopy-guided mucosal needle injection. (**A**) Overview of substances that can be delivered by needle-guided intra-colonic injection. (**B**) Illustration of colonoscopy technique. The endoscope is inserted into the rectum and the injection is performed in the mid-distal colon. (**C**) Set-up for the colonoscopy-guided injection. (**D**) Set-up of endoscope probe with injection needle. (**E**) Colonoscopy images showing the injection procedure. The needle is placed against the colon wall before the splenic flexure and the injection is applied into the mucosa. (**F**) Illustration of mucosal needle injection of substances from A. The injection is performed directly under the mucosa. (**G**) Schematic representation of tumor development and progression in the colon.

The fast and reproducible generation of orthotopic tumors in the rectum or colon of mice is of high importance to enable anatomical and pathophysiological reproduction of human CRC. To establish tumors in the colon, different substances or cells can be used and applied via colonoscopy-guided needle injection. Efficient tumor generation can be achieved via the injection of patient-derived organoids (PDOs), MDOs, or conventional 2D cell lines [16,29,30]. Furthermore, virus particles can be used for localized recombination when containing the coding sequence for Cre recombinase [12,31]. Additionally, the focal injection of 4-OHT leads to colon tumor formation when a *villin*Cre^ER^ background is used (Figure 1A,B) [13,15,32]. In this study, colonoscopy-guided injections could be performed on a benchtop or under a biosafety hood, depending on the specific application and local regulations. Besides the endoscopy tower containing the light source, air pump, camera system, and image processor, only a monitor, an aspirator for the inhalation of anesthesia, and a micro syringe were needed (Figure 1C). The endoscopic probe consisted of a needle shaft that permits the fine adjustment of the needle during the injection procedure, which was essential for precisely controlled injections (Figure 1D). The procedure was fast (<10 min) and straightforward. After the removal of feces by retrograde colonic lavage with PBS, needle-guided injection into the mucosa was carried out within two minutes (Figure 1E,F; Supplemental Movies S1–S3). Upon injection of compounds outlined above, tumor establishment and progression could be monitored longitudinally (Figure 1G) [12]. This technique generated local and anatomically defined tumors in the mid- or distal-colon of mice. The versatile applications described here represent a strong asset and advancement to the currently available preclinical toolbox of CRC modeling.

### 3.2. Generation of Autochthonous Colonic Tumors

**Figure 2:**
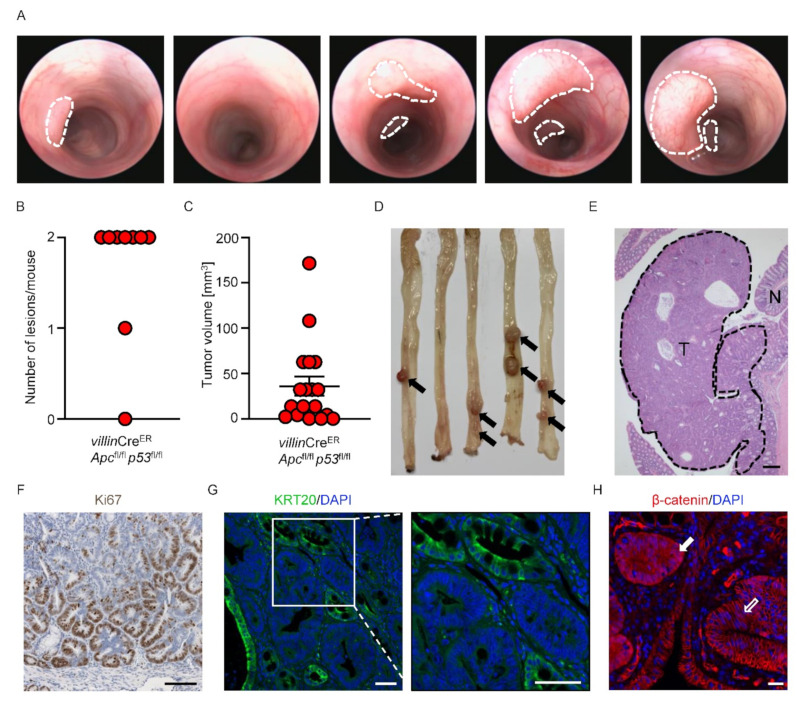
Colonoscopy-guided generation of locally defined tumors in a genetic model. (**A**) Representative colonoscopy images. Dashed lines mark the tumors. (**B**,**C**) Quantification of (**B**) number of tumors and (**C**) tumor volume detected 6 weeks after injection. (*n* = 9 mice). (**D**) Representative images of the colon in the same order as the colonoscopy images in (**A**). Arrows indicate the macroscopically visible tumors. (**E**) Representative H&E images of a colonic tumor. Dashed line marks tumor area (T) and N the normal mucosa. Scale bar indicates 200 µm. (**F**) Representative immunohistochemistry image of Ki67 on a *villin*Cre^ER^ *Apc*^fl/fl^ *p53*^fl/fl^ tumor. Scale bar indicates 100 µm. (**G**) Representative immunofluorescence images of KRT20 (green) on a *villin*Cre^ER^ *Apc*^fl/fl^ *p53*^fl/fl^ tumor. Scale bars indicate 50 µm. (**H**) Representative immunofluorescence image of β-catenin (red) on a *villin*Cre^ER^ *Apc*^fl/fl^ *p53*^fl/fl^ tumor. The empty arrow marks membranous and the filled arrow marks nuclear localized β-catenin. Scale bar indicates 20 µm.

The anatomically precise generation of autochthonous genetically engineered tumors is of importance when modelling CRC. To this end, we designed a strategy that allows for local recombination of driver genes that are frequently altered in CRC. Intercrossing of a *villin*Cre^ER^ transgene specifically led to recombination in intestinal enterocytes and triggered floxing of loxP flanked *Apc* and *Trp53* (*p53*). The localized injection of 4-OHT into the mucosa of the colon of *villin*Cre^ER^ *Apc*^fl/fl^ *p53*^fl/fl^ mice led to rapid tumor development within two weeks (Figure 2A). Two distinct injections per colon led to tumor establishment in >80% (15 tumors out of 18 injections) (Figure 2A–D). Histopathological analysis showed well-differentiated tubular adenomas with no detectable invasion, reminiscent of human adenoma (Figure 2E). Further, the expression of the proliferation marker Ki67, Keratin 20 (KRT20) as a marker of differentiated tumor cells, or nuclear β-catenin that reports WNT signaling activity, highlights cellular heterogeneity with varying degrees of differentiation within the tumors (Figure 2F–H). Together, this model recapitulated the key features of benign colonic tumors in a fully immunocompetent environment, allowing fundamental questions of tumor initiation and drug response to be addressed.

### 3.3. Orthotopic Mouse-Derived Organoid Transplantation

**Figure 3:**
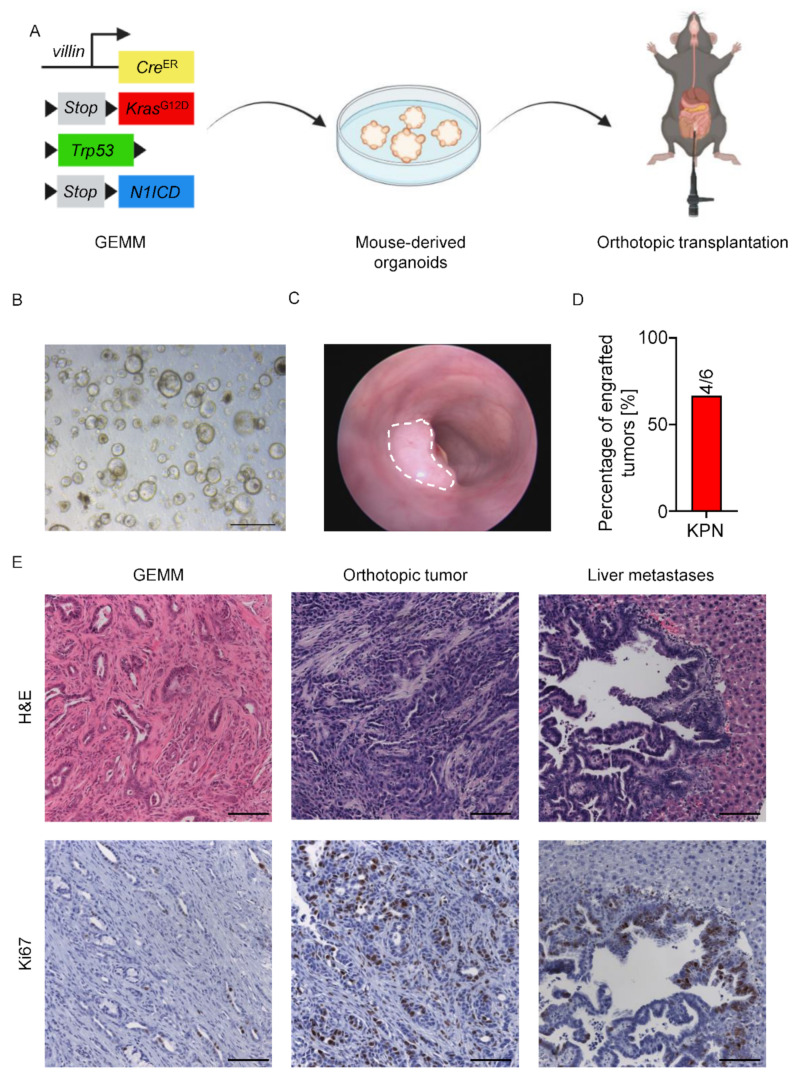
MDO generation and orthotopic transplantation. (**A**) Schematic representation of the generation of tumor-derived organoids from GEMMs and orthotopic transplantation of those MDOs. (**B**) Bright field image of KPN MDOs. Scale bar indicates 500 µm. (**C**) Representative colonoscopy image of a tumor derived from KPN MDOs two weeks post transplantation. (**D**) Percentage of engrafted tumors from KPN MDOs. (**E**) Representative H&E (top) and Ki67 (bottom) images of KPN GEMM, orthotopic tumors, and liver metastases originated from the orthotopic tumor. Scale bars indicate 100 µm.

Besides the recombination triggered by locally injected 4-OHT, we set out to perform MDO transplantation into the mucosa of immunocompetent C57BL/6 mice. This application allowed intra-tumor heterogeneity of advanced CRC to be investigated, including the tumor immune microenvironment. To this end, we generated MDOs derived from KPN GEMM tumors for subsequent intra-colonic injection (Figure 3A,B). Two weeks after the intra-colonic injection, KPN MDOs formed macroscopically detectable tumors (Figure 3C,D). Importantly, histological analysis of transplanted tumors and matched liver metastases showed poor differentiation and significant stromal components reminiscent of original GEMM tumors (Figure 3E). Taken together, this organoid transplantation model represents all characteristics of advanced CRC that are associated with poor prognosis.

### 3.4. Modeling Liver-Specific Metastasis

**Figure 4:**
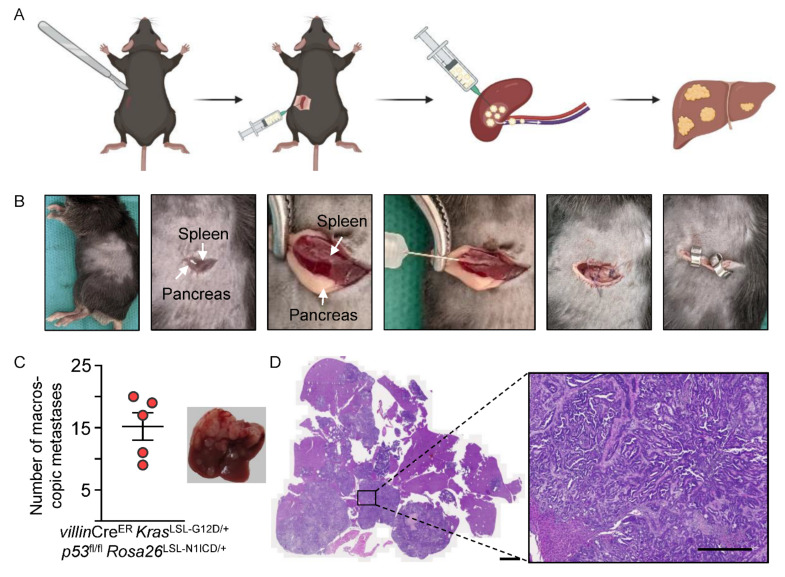
Modeling CRC liver metastasis by intrasplenic injection. (**A**) Illustration of the intrasplenic injection procedure of MDOs. (**B**) Images of the experimental set-up to model liver metastasis. (**C**) Left: quantification of the number of macroscopic KPN liver metastases nodules per mouse (*n* = 5). Right: representative image of liver metastases. (**D**) H&E images of KPN liver metastases. Scale bars indicate 2000 µm (left) and 500 µm (right).

The models described so far enable the precise and rapid generation of primary tumors in the colon. Importantly, the orthotopic transplantation of organoids into the colonic mucosa generates metastases in liver and lymph nodes [16]. To investigate metastasis in a setting reminiscent of patients after primary tumor resection and to study the later events of the metastatic cascade, we generated a model of hepatic metastasis. We injected single cells of KPN MDOs into the spleen of immunocompetent C57BL/6 mice (Figure 4A,B). A minimal left lateral axillary flank incision in the skin and subsequently in the peritoneal wall gave access to the spleen. Following that, the injection of MDOs directly into the splenic parenchyma led to metastases formation (Figure 4C). In this process, cells migrated into the liver through the portal venous system, enabling tumor cells to extravasate and form liver metastases. With this assay, a significant metastatic burden was generated (Figure 4C,D). This allows, for example, testing the effects of chemotherapies on established metastases, which is of strong clinical relevance. Additionally, extravasation, liver colonization, and establishment of macro-metastases could be investigated with this assay in a rapid and reproducible way.

### 3.5. Optical Barcoding Reveals Niche-Dependent Clonal Selection

**Figure 5:**
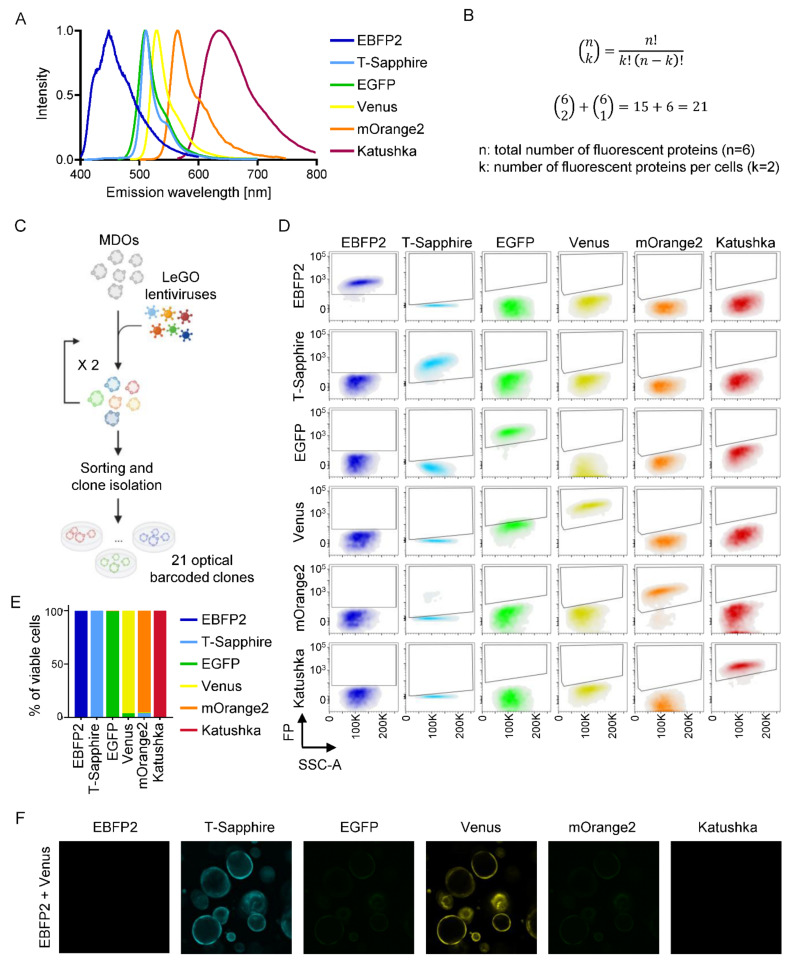
Optical barcoding to study clonal dynamics in CRC. (**A**) Histogram representing the emission spectra of the six different fluorescent proteins. (**B**) Binomial coefficient calculation to obtain the total number of clones that can be uniquely labeled with the LeGO optical barcoding system combining up to two colors per cell. (**C**) Schematic representation of the optical barcoding procedure of KPN organoids. (**D**) Flow cytometry plots of clones barcoded with one fluorescent protein. Each row corresponds to one clone. (**E**) Percentage of the fluorescence color detected by flow cytometry for the samples shown in (**D**). (**F**) Confocal images of the clone labelled with T-Sapphire and Venus fluorescent proteins.

**Figure 6:**
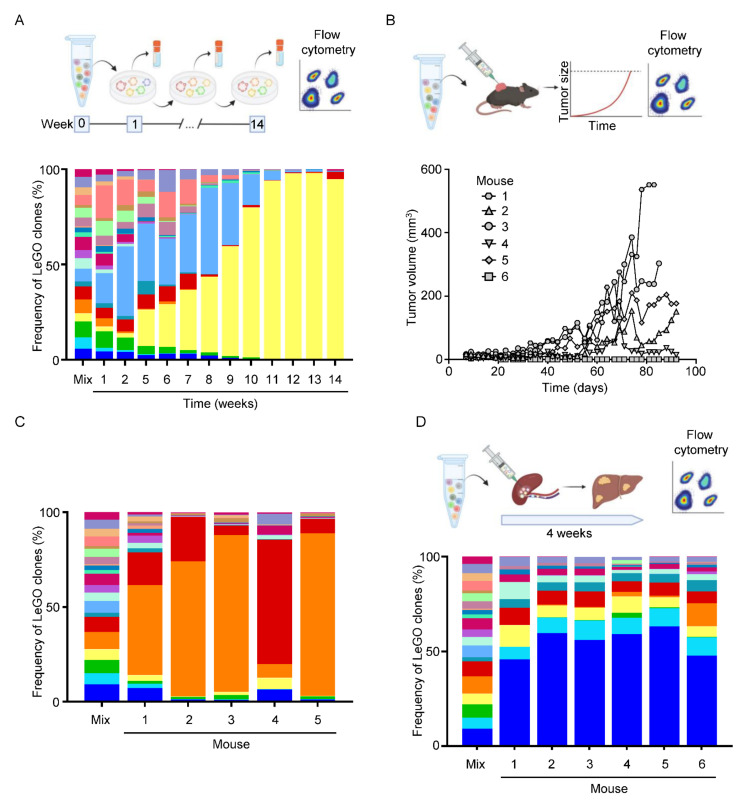
Clonal analysis for niche dependencies. (**A**) Schematic representation of the workflow for the study of clonal selection in vitro and the frequency of clones at indicated time points of the in vitro assay analyzed by flow cytometry. (**B**) Schematic representation of the workflow to study clonal selection in sub-cutaneous tumors and tumor growth of the sub-cutaneous tumors. (**C**) Frequency of the barcoded clones in sub-cutaneous tumors. (**D**) Schematic representation of the workflow for the study of clonal selection in liver metastases and frequency of barcoded clones in liver metastases detected by flow cytometry.

Emerging evidence indicates that tumor heterogeneity significantly contributes to cancer progression and therapy-resistance [33]. However, the complexity of the clonal composition is reduced during metastasis and treatment [34,35,36]. To understand the niche dependency during clonal selection, we implemented an optical barcoding strategy [37], by which six different fluorescent proteins were expressed in KPN MDO clones. To achieve individual optical barcoding of each clone, we infected cells with lentiviruses encoding for one or two of the fluorescent proteins (Figure 5A–C). With this strategy, we generated 21 individual clones that were distinguishable via flow cytometry (Figure 5D,E; Supplemental Figures S1A,B and S2) or immunofluorescence (Figure 5F). First, we wanted to understand whether clonal selection occurs in vitro. To this end, we seeded the 21 clones at similar ratios into 3D matrix and consecutively collected cells (Figure 6A). After 14 weeks, the clonal composition was analyzed by flow cytometry, which revealed that two clones (yellow and light blue) started to dominate the culture after ~5 weeks. At the end of the experiment, one clone (yellow) outcompeted the rest (Figure 6A). Notably, both dominating clones were not overrepresented at the beginning of the experiment, demonstrating that clonal selection is present under in vitro culture conditions.

Next, we set out to define potential clonal selection in vivo. First, we performed subcutaneous injections of an equal mixture of the 21 clones. Longitudinal tumor monitoring showed slow establishment of five out of six tumors (Figure 6B). Interestingly, we detected a different dominating clone (orange) compared to in vitro (Figure 6C). Notably, one tumor showed prevalence of a clone (red) that was not detected in any of the other mice from this experiment, potentially due to a reduced tumor diameter at endpoint. To study the clonal selection in metastases, we performed the aforementioned intrasplenic injection with the 21 clones mixed at equal ratio to test colonization and outgrowth in the liver (Figure 6D). In contrast to in vitro and sub-cutaneous, we detected another dominating clone (Figure 6D). Interestingly, an overall clonal heterogeneity was still detectable at this time point. Together, we show that the environmental niche significantly influences clonal selection.

We also performed intra-colonic injection of the 21 clones into the mucosa as described above to understand the clonal selection in the colon. However, in 12 mice (out of two independent experiments), no tumor establishment was detectable, showing that none of the 21 clones had the ability to grow successfully in the colon. Since parental KPN organoids showed growth in the colon (Figure 3C,D), we speculated that the expression of fluorescent proteins potentially attenuates engraftment. To test this, we used lentivirus-mediated gene delivery for the constitutive expression of luciferase 2 (luc2) and tdTomato in an additional MDO model (Supplemental Figure S3A,B). MDOs were derived from GEMMs harboring alterations in the tumor suppressor *Apc* (*Apc^f^*^l/fl^), the oncogene *Kras* (*Kras*^G12D/+^), the tumor suppressor *Trp53* (*p53*^fl/fl^), and the TGF-β pathway (*Alk5*/Tgfbr1^fl/fl^) (AKPT) [16]. Three rounds of tdTomato-positive AKPT cell enrichment followed by clonal isolation generated a stable tdTomato-positive cell pool (Supplemental Figure S3A,B). Colonoscopy monitoring at two weeks after mucosal organoid injection showed reduced tumor formation of tdTomato-expressing cells (clone #1 1/10; clone #2 2/5) (Supplemental Figure S3C,D). Similarly, a localized signal in the abdomen was detected by bioluminescence imaging two weeks post injection, which was only detected in the animals with confirmed tumors. To exclude an engraftment defect of the isolated clones and to maintain intra-tumor heterogeneity of the MDO population, we injected the transduced MDOs as well as the polyclonal bulk population. This resulted in tumor formation only in 2 out of 12 mice (Supplemental Figure S3D). We also used non-transduced (parental) AKPT MDOs to rule out a potential immunogenic effect to the transduced cells. These parental MDOs were capable to form tumors in 80% (4/5) of the mice. Together, our data indicate an enhanced clonal selection in the colon, compared to the liver or sub-cutaneous, which is potentially driven by expression of fluorescently labeled cells transplanted to immunocompetent mice.

## 4. Discussion

Here, we described a versatile tool kit used to establish preclinical CRC models, enabling the generation of CRCs at different stages with high relevance for the human disease. Importantly, the colonoscopy-guided mucosal injection of CRC cells, organoids, virus, or other substances is fast and leads to robust results. Notably, this procedure was performed without invasive surgical manipulation, which significantly improved animal welfare. These methods represent a highly reproducible approach to generate CRC liver metastases. Depending on the type of induction and route, the most relevant sites for primary tumors and metastasis were modeled.

The ability to generate primary- or secondary-site CRC in the correct anatomical location with a high tumor uptake is crucial for cancer research. Here, we described the induction of tumors harboring different genetic backgrounds, thus permitting modeling of relevant CRC subtypes. The colonoscopy-guided injection allows tumor organoids with various genetic alterations to be implanted into immunocompetent or immunodeficient mice. We used AKPT organoids which resemble the classical route of CRC or KPN organoids to model the less characterized serrated route [16], showing that models of different CRC subtypes can be employed with these techniques. Besides this, complex driver mutation combinations can be introduced to PDOs or MDOs by gene editing of organoids. The only limitation for organoids might be a reduced engraftment rate of early-stage or low driver mutation burden tumors. However, the injection of 4-OHT into mice harboring alterations, for example in *Apc* and *Trp53*, showed the rapid establishment and growth of well-differentiated adenomas. Therefore, the colonoscopy-guided injection enables the generation of early-stage adenomas, invasive metastatic carcinomas, as well as different CRC subtypes. The approach described here shows a high engraftment rate with ~80% for intra-colonic injections and 100% in the intrasplenic model. Recent studies suggested the usage of NOD SCID gamma (NSG) mice to increase the success rate of organoid injections [12]; however, this comes with the caveat of the loss of an intact immune system.

Human CRC is believed to be initiated by one mutated cell; nevertheless, the localized injection of 4-OHT will activate the Cre-mediated recombination in multiple crypts. To our knowledge, the targeting of single cells for recombination has not been tested by 4-OHT dilution to date and might be beneficial to study clonal evolution of tumors. Various technologies have been established to model CRC with localized disease. One technique that can be compared to the approach presented here is the generation of a rectal prolapse to inject organoids; notably, this application is feasible without special equipment [38,39,40]. However, this technique restricts injections to the rectal area. Further, a commonly applied model to resemble intestinal tumors is the transplantation of cancer cells into the cecum by sub-serosal injection or installation of collagen plaques that contain organoids [40,41]. Interaction with the intestinal microbiome, which plays important roles in CRC progression, therapy response, and metastasis, is critical [42]. Hence, the anatomical difference between the murine cecum and the human appendix and further the microbial and metabolic differences between cecum and colon might generate confounding components when using the cecal injection model. In addition, the segmental application of virus-encoding Cre recombinases, for example, have been demonstrated as a robust tool [43]. This tool compared to the rectal prolapse or colonoscopy-guided injection requires a surgical laparotomy; hence, this technique might suffer from surgical complications in its efficiency, requires an increased technical competence, and is time-consuming. Alternatively, normal intestinal epithelial organoids, PDOs or MDOs, can be installed into the colon after mechanical or chemical damaging epithelial integrity with ethylenedinitrilotetraacetic acid (EDTA) in combination with brush-mediated epithelial abrasions [44,45] or dextran sodium sulfate (DSS)-induced injury [46,47,48,49]. The implantation of cancer organoids after DSS treatment also leads to submucosal tumor formation; however, as this treatment damages the whole colon, tumor cells can integrate at multiple sites, potentially leading to a vast number of tumors and a huge tumor burden. As the overall aim is to resemble the human pathology as closely as possible, this approach, by generating multifocal lesions, bears disadvantages.

Future technical improvements should enable the modeling of right-sided tumors with needle-guided injections. This might be realized with the use of flexible endoscopy probes and will reduce pan organ recombination effects induced by systemic tamoxifen injections. The local administration of tumor cells, organoids, or virus particles that lead to a single tumor could also be the base for further important developments. The use of specific virus particles harboring the CRISPR/Cas9 system to generate deletions and mutations of CRC driver genes could be extended for genetic screening approaches. More relevant to recapitulate the clinical setting is that a single tumor in the colon is suitable for surgical resection. Indeed, such models would resemble the human treatment regimen and enable the study of metastatic recurrence or tumor cell dormancy.

Generating metastatic CRC models is crucial for our understanding of late-stage CRC, which is the main cause of CRC mortality. Tail–vein injections to generate lung metastases are established for CRC [50]; however, for CRC, the primary metastatic organ is the liver [51]. Thus, modeling liver metastasis is of high importance to understand mechanisms driving progression. The colonoscopy-guided injection of organoids into the colon has the ability to generate liver metastases, with a metastatic frequency depending on the genetic background of the organoids. *Apc*-deficient organoids were not able to form liver metastases, whereas organoids derived from *Apc^f^*^l/fl^, *Kras*^G12D/+^, and *Trp53*^fl/fl^ (AKP) mice or engineered organoids resembling the same genetic alterations developed metastases in one third or in one sixth of the mice after 12 or 16 weeks, respectively [12,49]. To model CRC liver metastasis, the injection of organoids into the spleen, as described, leads to liver metastases with high efficiency. The reported portal venous injection procedure generates liver metastases at a comparable burden [38]; however, a caveat of this approach is that a major abdominal vessel is punctured during the procedure, and this comes with high risk of mortality due to leakage. Therefore, the intrasplenic model represents a strong option to circumvent complications, such as the rupture of the portal vein or liver artery, and requires less training. The ectopic injection of CRC tumor cells into the kidney capsule or sub-cutaneous into the flank leads to rapidly growing tumors that are easy to be observed longitudinally [22,23]. Since the TME plays an essential role in tumor establishment, progression, and therapy resistance [52], these sites might influence tumor biology and do not recapitulate important details of human tumors. For example, those sites display remarkable differences in blood vessel supply and lymphatic drainage compared to the colon. Eventually this leads to dissimilar metastasis, compared to the usual route of CRC tumor cells that travel via the portal venous system to the liver; hence, the orthotopic model presented here resembles the human scenario much closer [53]. This is also supported by our analysis of the clonal selection.

Intra-tumor heterogeneity is believed to be causative for the progression and therapy resistance of CRC [54,55], which should be recapitulated by preclinical models to the best extent. The approaches presented here demonstrate the use of different driver mutations, thus represent the inter-tumor heterogeneity observed in patients. Notably, further expansion of additional subtypes is feasible. The heterogeneous expression of KRT20 and WNT signaling activity demonstrates a high degree of intra-tumor heterogeneity present in these tumors. Together, intra- and inter-patient heterogeneity can be faithfully modeled with these systems.

To understand the clonal selection during CRC progression, we employed an optical labelling system that allows longitudinal tracing of individual clones at different sites. Similar to previous studies in mouse models of pancreatic cancer, breast cancer, and squamous cell carcinoma [56,57,58], we determined that clonal selection is a dominant event during cancer progression. Importantly, we demonstrate that clones, which expand under in vitro conditions, differ to in vivo selected clones. This implicates a critical bottleneck during in vivo growth, intriguingly independent of the common in vitro starting culture. Our results demonstrate that clonal selection is niche-dependent, leading to speculations on the importance of individual TMEs selecting clones with the highest engraftment or seeding efficiency. Similar clonal selection was observed in breast cancer after transplantation of human fluorescently labelled cell lines into immune-deficient NSG mice [56]. In this study, lung metastases were polyclonal and liver metastases monoclonal. In another study, neo-antigen level modeling in CRC organoids and syngeneic transplantation showed that already low-level neo-antigen expression leads to T cell dysfunction [59], in low mutational burden CRC models. Critically, tumor rejection was only observed in tumors with high neo-antigen expression [59]. Our results indicate a potential role of the expression of fluorescent proteins during clonal selection at least in the colon. The rejection of tumors in the colon indicates increased immune surveillance compared to liver and sub-cutaneous tumors. This is in line with recently reported restrained primary tumor growth and metastasis as a result of increased immunogenicity against GFP in breast cancer metastasis models [60].

The clonal labelling of CRC cell lines demonstrated that the positioning of a clone is more critical for its outgrowth than cell intrinsic signals [61,62]. This was particularly evident at the tumor edge, alluding to the supply of factors at the stroma tumor interface. This shows that micro-environmental components contribute essential signals to support clonal expansion [63]. Our results are in line with this observation when it comes to the micro-environmental impact on clonal selection. However, previous studies utilized immunocompromised mice, which might explain the stronger clonal selection observed in our syngeneic models. Together, these observations demonstrate a fundamental role of the TME on clonal selection and tumor growth.

## 5. Conclusions

The modeling approaches presented here are straightforward tools to generate CRC at various stages of progression, from adenomas to locally advanced tumors to metastasis. By utilizing these models, we demonstrate that niche-dependent clonal selection of CRC in varying TMEs has potential implication for future therapeutic strategies.

## Figures and Tables

**Table 1:** Configuration of the BD FACS Aria Fusion 1.

Fluorescent Protein	Laser (nm)	Dicroic Mirror (nm LP)	Bandpass Filter (nm)
EBFP2	405	-	450/50
T-Sapphire	405	505	530/30
EGFP	488	505	510/20
Venus	488	550	542/27
mOrange2/tdTomato	561	570	582/15
dKatushka	561	735	660/20

**Table 2:** Configuration of the BD LSR Fortessa.

Fluorescent Protein	Laser (nm)	Dicroic Mirror (nm LP)	Bandpass Filter (nm)
EBFP2	405	-	450/50
T-Sapphire	405	505	525/50
EGFP	488	505	515/20
Venus	488	550	560/40
mOrange2/tdTomato	561	570	586/10
dKatushka	561	735	780/60
* ZombieNIR	640	750	780/60

* Zombie NIR was used to assess cell viability.

## Data Availability

The data presented in this study are available in this article.

## Supplementary Materials

The following supporting information can be downloaded at: https://www.mdpi.com/article/10.3390/cancers14174260/s1. Supplemental Movie S1: Flushing. Removal of feces by retrograde colonic lavage using PBS. Supplemental Movie S2: Injection-handling. The endoscope is carefully inserted into the rectum and positioned at the mid-distal colon, where the injection takes place. Supplemental Movie S3: Injection-intracolonic. The needle is carefully placed against the colon wall and the injection is performed. Figure S1: Characterization of barcoded clones. (A) Flow cytometry plots of the double-colored barcoded clones. (B) Table containing the fluorescent barcode of the 21 different clones and the color code used in Figure 6. Figure S2: Representative image of the flow cytometry plots obtained from a mix of the 21 KPN tumors. Figure S3: MDO modification and orthotopic transplantation. (A) Illustration of AKPT Luc2/tdTomato expressing organoid generation and enrichment by FACS with subsequent injection. (B) Bright field and fluorescent images of AKPT organoids. Scale bars indicate 200 µm. (C) Representative colonoscopy images showing no tumor (left) and a tumor that developed after mucosal injection of AKPT organoids (right). (D) Percentage of engrafted tumors from the orthotopic transplantation of parental and transduced AKPT MDOs. (E–G) Representative H&E images of (E) AKPT MDOs, (F) AKPT orthotopic tumor, and (G) spontaneous AKPT liver metastases derived from the orthotopic tumor. Scale bars indicate 200 µm.
