# Supplementary material for: Modeling Colorectal Cancer Progression Reveals Niche-Dependent Clonal Selection
Source: Cancers (Basel). 2022 Aug 31;14(17):4260. doi: 10.3390/cancers14174260 (PMC9454531; doi:10.3390/cancers14174260)

Supplemental Figure S1

A

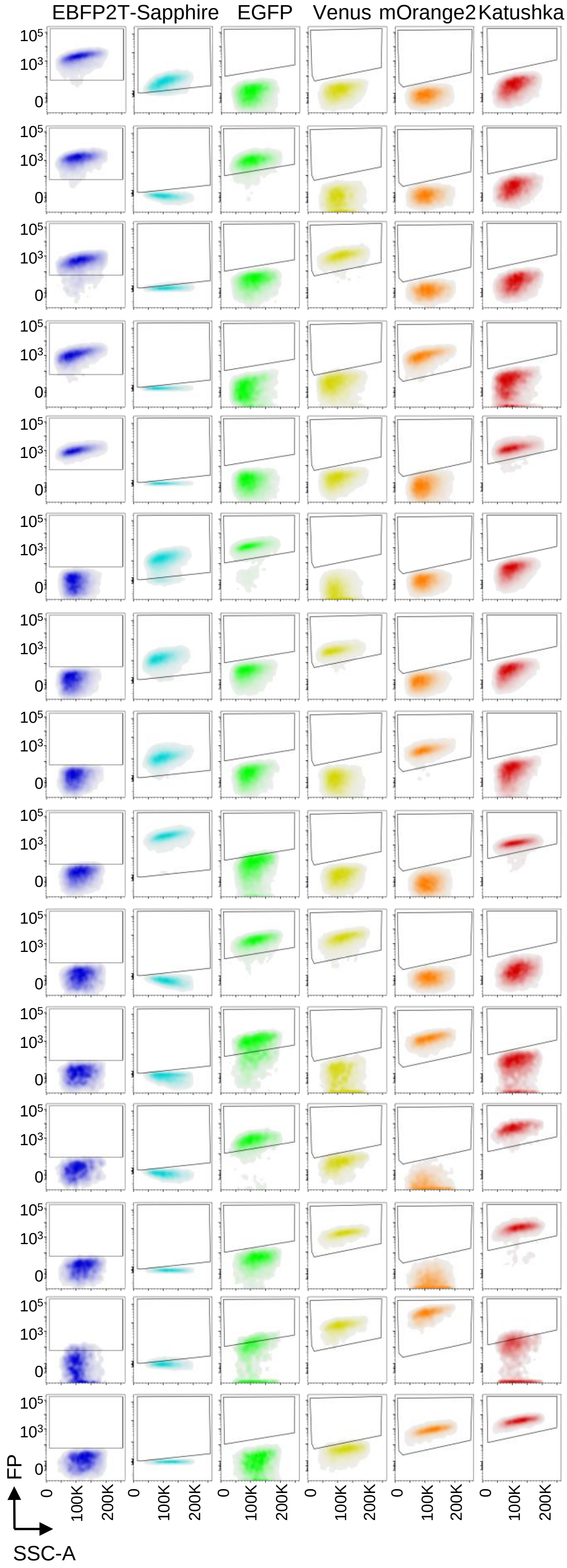

B

Clone ID	Color code	Fluorescent protein composition
1		EBFP2
2		T-Sapphire
3		EGFP
4		Venus
5		mOrange2
6		Katushka
7		EBFP2 + T-Sapphire
8		EBFP2 + EGFP
9		EBFP2 + Venus
10		EBFP2 + mOrange2
11		EBFP2 + Katushka
12		T-Sapphire + EGFP
13		T-Sapphire + Venus
14		T-Sapphire + mOrange2
15		T-Sapphire + Katushka
16		EGFP + Venus
17		EGFP + mOrange2
18		EGFP + Katushka
19		Venus + mOrange2
20		Venus + Katushka
21		mOrange2 + Katushka

Supplemental Figure S2

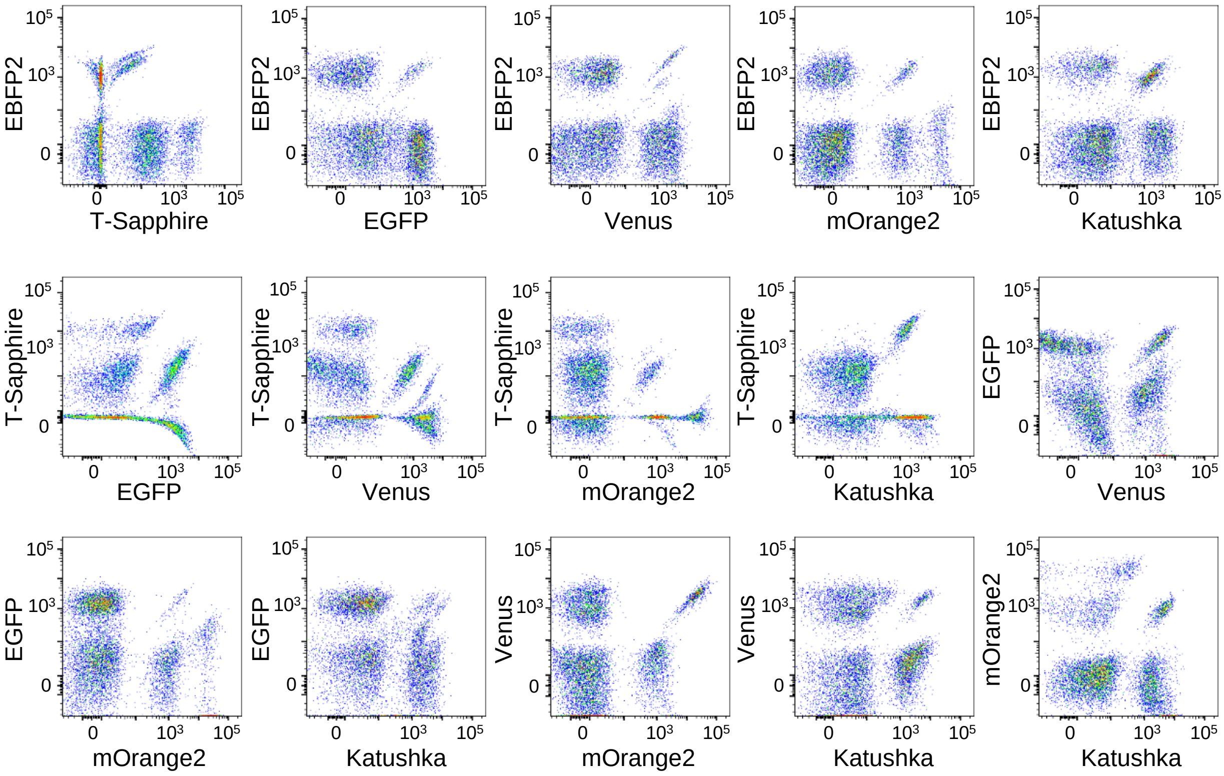

Supplemental Figure S3

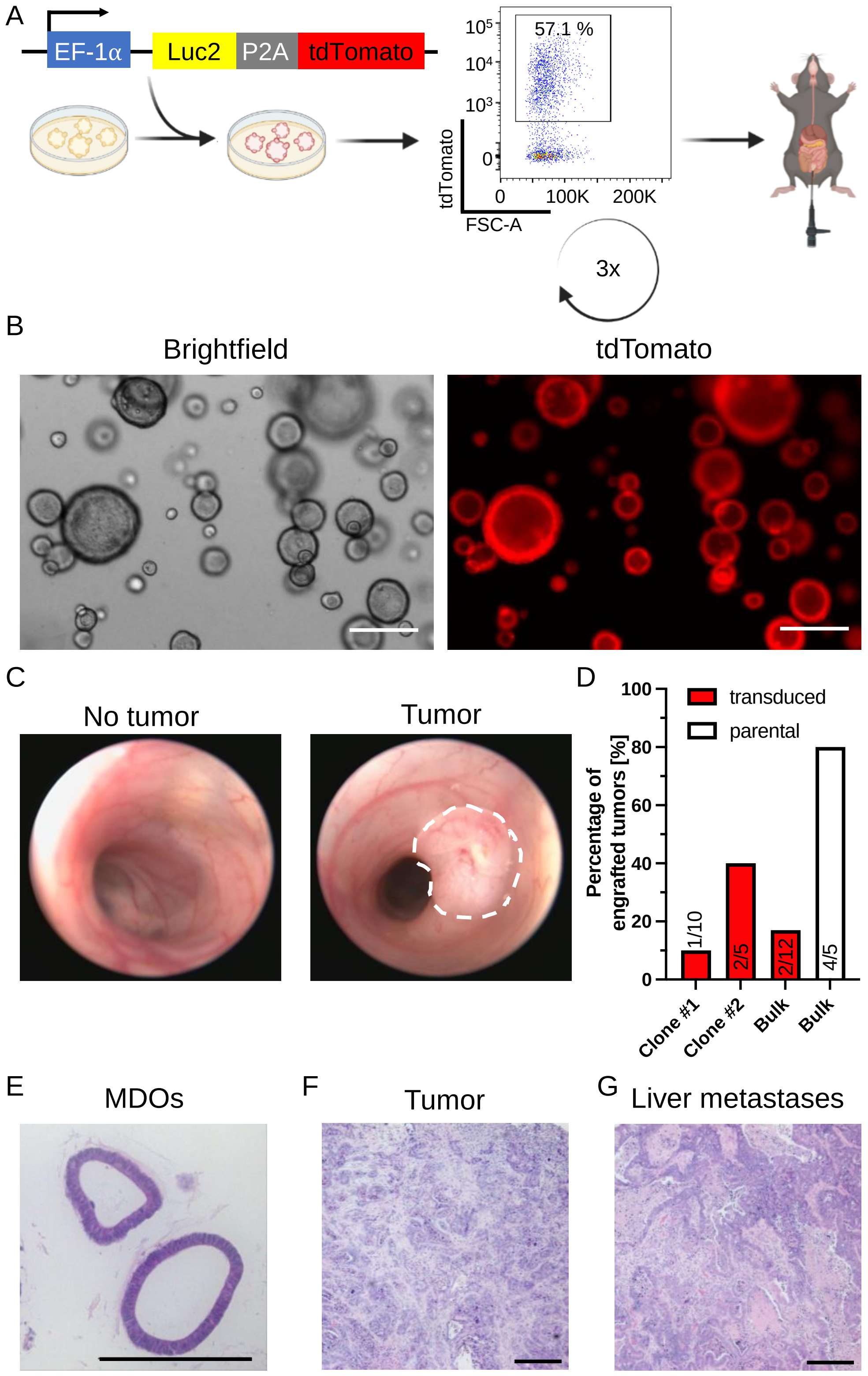

Supplement: Supplementary file 1 [file cancers-14-04260-s001.zip › cancers-1872384-supplementary.pdf]
